# Celestial Moderation of Tropical Seabird Behavior

**DOI:** 10.1371/journal.pone.0027663

**Published:** 2011-11-14

**Authors:** Patrick Pinet, Audrey Jaeger, Emmanuel Cordier, Gaël Potin, Matthieu Le Corre

**Affiliations:** Laboratoire d'Ecologie Marine, Université de La Réunion, Saint Denis, Ile de La Réunion, France; Monash University, Australia

## Abstract

Most animals, including birds, have cyclic life histories and numerous studies generally conducted on captive animals have shown that photoperiod is the main factor influencing this periodicity. Moon cycles can also affect periodic behavior of birds. Few studies have investigated the influence of these environmental cues in natural settings, and particularly in tropical areas where the change in photoperiod is slight and some bird species keep cyclic behaviors. Using miniaturized light sensors, we simultaneously investigated under natural conditions the influence of photoperiod and moon phases on the migration dates and at-sea activity of a tropical seabird species, the Barau's petrel, throughout its annual cycle. Firstly, we found that birds consistently started their pre- and post-breeding migrations at precise dates corresponding in both cases to a day-duration of 12.5 hours, suggesting a strong influence of the photoperiod in the regulation of migration behavior. We also found that mean population arrival dates to the colony changed from year to year and they were influenced by moon phases. Returns at their colonies occurred around the last full moon of the austral winter, suggesting that moon cycle is used by birds to synchronize their arrival. Secondly, variations of day-time activity were sinusoidal and correlated to seasonal changes of daylength. We thus hypothesize that the photoperiod could directly affect the behavior of the birds at sea. Night-time at-sea activity exhibited a clear cycle of 29.2 days, suggesting that nocturnal foraging was highly regulated by moon phase, particularly during the non-breeding season. To our knowledge, this is the first study to document a mixed regulation of the behavior of a wild bird by photoperiod and moon phases throughout its annual cycle.

## Introduction

The annual cycle of birds comprises life-history stages of breeding, molt and, for numerous species, migration. Varying environmental conditions determine the optimal period for each stage and their timing has major consequences for fitness [1]–[3]. Birds rely on both endogenous clock and predictable environmental cues to indicate the time of year and schedule their life-history stages [4]. They can maintain over a wide range of period length their circannual rhythm under constant captive conditions through internal clocks but require a seasonal signal for synchronicity over the long-term [4]. Because seasonal change in photoperiod is entirely predictable between years, day-length has been adopted by various organisms, including birds, as the main environmental cue to match physiological processes and behavioral decisions with seasonal changes in environmental productivity [5]–[7]. To respond appropriately to short-term and unpredictable environmental changes, birds use secondary non-photoperiodic cues such as ambient temperature, rain, food supply or nest site availability [8]–[10]. Lunar cycle has also been shown to affect the biology and phenology of many animals, such as their migratory, reproductive or feeding behavior [11]–[15].

The degree to which environmental cues trigger bird physiology and behavior varies with latitudes. Because optimal environmental conditions for breeding occur only during a particular season each year, species of the temperate and polar zones exhibit highly predictive seasonal breeding and migration. Photoperiod in these latitudes is clearly different between seasons, and many studies have demonstrated that birds principally time their annual cycle with the annual change in day-length [10]. By contrast, the seasonal variations in photoperiod and in environmental productivity are slight in the tropical zone. However, many birds breeding within the tropics also have high seasonal predictable breeding and migration activities and their annual cycles are influenced by small scale variations in daylength [16]–[18]. It remains unknown how they adjust and synchronize their reproductive or migratory behavior in an environment with low seasonality and minor photoperiodic variations. In contrast to species from temperate and polar zones, the timing processes of wild birds inhabiting tropical environments, and particularly marine species, are poorly studied (but see [11], [18]).

Due to the large-scale and rapid movements of seabirds, their locations at sea and thus the environmental cues that could trigger their behavior during their annual cycle have been inaccessible in natural settings until recently. Seabird biologging has made many advances over the past twenty years enabling researchers to study at-sea ecology of free-ranging animals in their natural environments [19]. Recently developed Global Location Sensors (GLS) allow tracking of seabirds over the long-term of their annual cycle [20]–[22]. Tags record light referenced to time, then latitudes are calculated from day and night durations and longitude from the times of mid-day and mid-night [23], [24]. GLS also records wet/dry activity (immersion) providing information on seabird at-sea behavior (i.e. proportion of time on flight or resting on sea). Here we propose a novel use of GLS as a “photoperiod recorder”, not only to estimate geolocation, but also to measure the day-length perceived by birds along their at-sea movements to research the potential regulatory effect of day length on the behavior of the birds. The aim of the present study is to investigate under natural conditions, thanks to day-length measurements, geolocation and at-sea activity provided by GLS, the effect of two main environmental cues, the photoperiod and the moon cycles, on the behavior of a tropical seabird species. We thus studied the effects of (1) annual photoperiod cycle on day-time at-sea activity and date of migration and of (2) moon cycle on night-time at-sea activity and date of migration.

**Figure 1:**
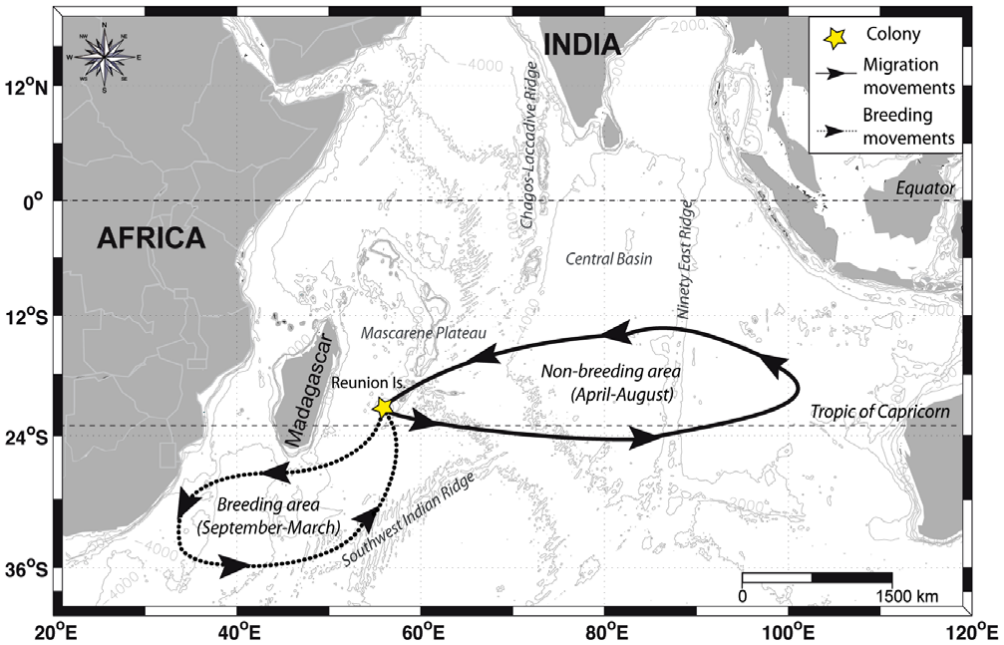
Breeding and non-breeding movements of Barau's petrels through their annual cycle. Information comes from at-sea surveys [63], [64] and recent tracking studies [27].

We chose the Barau's petrel (*Pterodroma baraui*), an endemic Indian ocean Procellariiforme [25] as seabird model because it exhibits a predictable annual timing of breeding and migration [26], [27]. This species also exhibits a clear segregation in its breeding and non-breeding locations (review in [28], Fig. 1). Finally, this species makes longitudinal migrations [27] although most seabirds perform latitudinal migrations [29], [30]. It thus remains in the tropical zone with slight photoperiodic variations during its whole life cycle. Barau's petrel represents therefore a relevant model to study the relationships between photoperiod and the behavior of tropical seabird species.

## Materials and Methods

### Field study

The study was carried out on Barau's petrels from February 2008 to February 2010 at Reunion Island in the western Indian Ocean (55.33°E and 21.07°S, Fig. 1). During the breeding season (September to April), Global Location Sensing loggers (GLS, Mk14, British Antarctic Survey) attached to metal rings with cable ties were deployed on the tarsus of 55 petrels, not simultaneously but at varying dates. Mass of logger, ring, and cable ties was 2.3 g, *i.e.* 0.6% of adult body mass (∼380 g), which is below the 3% limit recommended for flying birds [31], [32] and all birds were handled less than 10 minutes. Overall, the recovery rate was 81.8% (n = 45), four loggers recorded errors caused by problems of water incursion and one had been lost when the bird returned. 41 GLS loggers were thus successfully downloaded and between 25 and 247 days of data recording were obtained for each individual (mean = 100.6 days, SD = 58, n = 41). This study has been supported by the National Park of Réunion Island (Parc National des Hauts de La Réunion) and the French ringing center (Centre de Recherches par le Baguage des Populations d'Oiseaux, CRBPO, permit code: MLC) from the Museum National d'Histoire Naturelle, allowing us to manipulate birds at their colonies.

### Activity and light data

**Figure 2:**
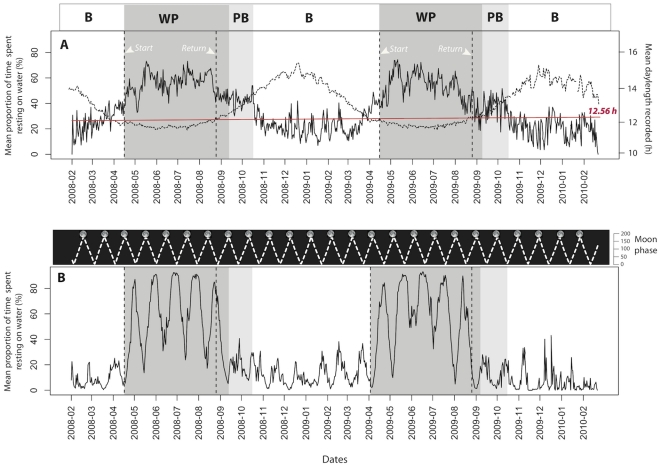
Mean proportion of time spent resting on water (i.e. at-sea activity) of Barau's petrels (N = 41) over two consecutive years during a) day-time and b) night-time according to mean day-length recorded (dashed line) and moon phase, respectively. Abbreviations report to Barau's petrel cycle of life: B, breeding period; WP, wintering period; PB, pre-breeding period. Red line represents the threshold day-length value where birds initiated their migration trips (see results).

The GLS loggers recorded immersion in seawater and light intensity with reference to time. Immersion in seawater was measured every 3 s and recorded as 0 or 1. These data were integrated during each 10 min recording period, thereby providing a value from 0 (no immersion during 10 min) to 200 (continuous immersion during 10 min). These ratios were then used as a proxy of the proportion of time spent on water during the 10 min period, called at-sea activity. A value of 0 indicated an active flying bird and a value of 200 an inactive bird entirely on water for 10 min. This ratio was calculated daily for each bird and then averaged for birds tracked at this date to obtain a time-series over two years (Fig. 2). We also calculated the total time of activity per day (in hours) to analyze the change of the time of activity under different photoperiod. Barau's petrels were equipped at varying dates over the two years and per day, the number of birds tracked ranged from 5 to 31. Light levels were measured at 60 s intervals and the maximum values during each 10 min period were logged, allowing estimations of locations depending on the sunset and sunrise [33]. Geographical locations of migration areas and trips derived from light time series are already published [27], and summarized in Figure 1. In the present study, light level records were used to calculate day-length received by birds and segregate the proportion of time spent on water per day-time and night-time. Indeed, many studies have shown clear differences in foraging behavior between night-time and day-time in Procellariiformes [20], [34]–[36].

### Data analysis

A spectral analysis was used to identify the dominant frequencies in night-time and day-time time series of Barau's petrel activity. The signal (the time series) is decomposed into harmonic components based on Fourier analysis. This can be regarded as a partition of the variance of the series into its different oscillating components with different frequencies (periods). Peaks in the periodogram or in the spectrum indicate which frequencies are contributing the most to the variance of the series. In this manner, periodicities, if present, are detected [37]. Relationships between environmental cues (day-length recorded and moon cycle downloaded from http://www.imcce.fr/fr/ephemerides/formulaire/form_ephepos.php) and seabird activity were tested with Pearson's correlations. The initiation dates of pre- and post-breeding migration of 23 birds (Table 1) were determined with the oriented trajectory method [38], [39] that allows determining whether birds actively oriented themselves towards a specific area (colony or wintering area). The arrival dates to the colony (*i.e.* the end of the pre-breeding migration) were identified at the first absence of light signals during day-time when birds were in burrows (Table 1). All analyses were performed with R (2.13.0) except the FFT accomplished using MATLAB®.

**Table 1:** Summary of day-length recorded by loggers and dates at the initiation of pre- and post-breeding migrations, and of arrivals dates to the colony of 23 Barau's petrels for two years.

Years	ID	Post-breeding migration dates	Day-length	Pre-breeding migration dates	Day-length	Arrival dates
**2008**	8090–X9	01/04/08	12.66	31/08/08	12.58	22/09/08
	8094– GBN21	23/03/08	12.65	23/08/08	12.49	19/09/08
	8095–F3	03/04/08	12.50	28/08/08	12.46	03/09/08
	8102–GBN2	16/04/08	12.37	18/08/08	12.46	11/09/08
	8104–GBN72	23/04/08	12.38	23/08/08	12.49	06/09/08
	8107–GBN26	20/04/08	12.33	29/08/08	12.53	20/09/08
	8114–GBN26	04/03/08	12.33	25/08/08	12.42	17/09/08
	8119–F1	15/03/08	12.66	23/08/08	12.59	24/09/08
	8120–X7	01/04/08	12.50	11/09/08	12.55	25/09/08
	8121–GBN3	17/04/08	12.66	27/08/08	12.52	06/09/08
	8126–A21	09/04/08	12.50	18/08/08	12.46	01/09/08
	8106–GBN5	02/04/08	12.50	03/09/08	12.58	09/09/08
	***MEAN***	***03/04/08***	***12.50***	***26/08/08***	***12.51***	***13/09/08***
	***SD***	***15.0***	***0.13***	***6.8***	***0.06***	***8.6***
**2009**	8114–MM4	10/04/09	12.50	26/08/09	12.50	07/09/09
	9150–F2	23/03/09	12.66	31/08/09	12.33	15/09/09
	8093–F3	24/03/09	12.70	21/08/09	12.67	26/08/09
	8121–GBN26	17/04/09	12.40	09/09/09	12.50	14/09/09
	8108–GBN72	03/04/09	12.80	25/08/09	12.33	01/09/09
	8095–GBNT1	03/04/09	12.73	18/08/09	12.67	25/08/09
	8123–GBNT1	05/04/09	12.83	25/08/09	12.50	02/09/09
	8111–MM4	24/03/09	12.50	29/08/09	12.83	03/09/09
	8105–MM14	11/04/09	12.50	23/08/09	12.67	03/09/09
	8102–GBN54	04/04/09	12.50	29/08/09	12.67	07/09/09
	8133-GBN46	29/03/09	12.83	18/08/09	12.50	01/09/09
	***MEAN***	***02/04/09***	***12.63***	***25/08/09***	***12.56***	***03/09/09***
	***SD***	***8.1***	***0.16***	***6.3***	***0.15***	***6.7***

## Results

### Day-time activity, migration dates and daylength

**Figure 3:**
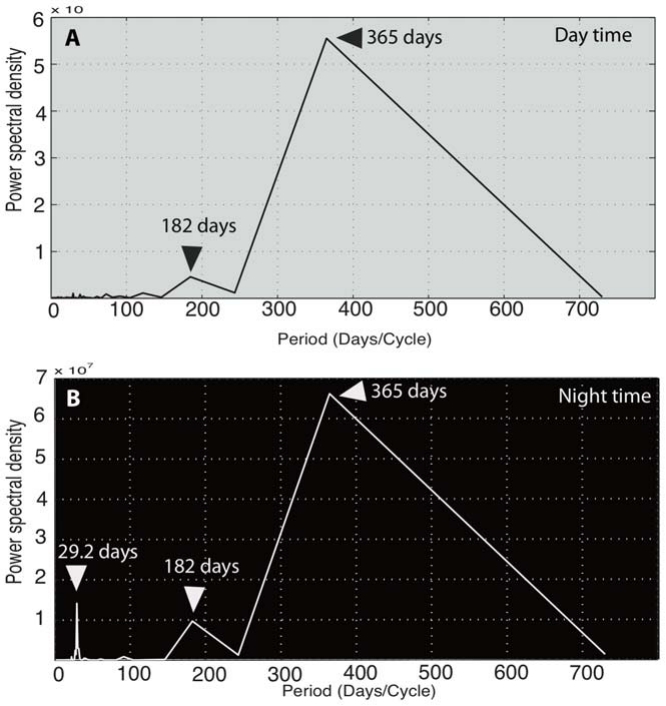
Periodogram for (a) day-time activity and (b) night-time activity. Each peak represents a period contributing significantly to the total variance of the signal.

About two hundred thousand hours (from 5^th^ of February 2008 to 25^th^ of February 2010) of continuous high-resolution (10 min) activity and light data were recorded. The proportion of time spent on water (at-sea activity) recorded during day-time over the whole studying period followed specific cycles (Fig. 2a). The spectral analysis showed two peaks at 182 (i.e. 6 months) and 365 days (i.e. 1 year) (Fig. 3a). Theses frequencies revealed that at-sea activity followed an annual cycle divided into two equal seasonal parts (i.e. breeding and non-breeding) and repeatedly reproduced each year. Barau's petrels were more active (i.e. not resting at sea) while breeding than during the non-breeding season, they spent respectively a daily mean of 26.6±12.2% (mean total activity: 2.7 h ±1.3 h) and 53.1±10.5% (6.8 h ±1.1 h) resting on water (Fig 2a, *t*_747_ =  −31.01, P<0.005). The day-length recorded ranges from 11.2 h to 15.6 h and showed significant differences between breeding and non-breeding periods (13.96±0.71 h and 12.32±0.16 h, respectively, *t*_747_ = 39.93, P<0.005). Importantly, we found a negative correlation between day-length and day-time activity (Fig 2a, *Pearson*'*s correlation, r* =  0.74, df = 750, P<0.005).

Despite slight inter-individual differences, birds consistently started their post-breeding migration to reach their wintering areas in early April for both years (Table 1, mean departure date in 2008: 3^rd^ of April ±15.0 days and in 2009: 2^nd^ of April ±8.1 days). Similarly, they consistently left their wintering area for their pre-breeding migration in late August for both years (Table 1, mean return date in 2008: 26^th^ of August ±6 days and 2009: 25^th^ of August ±6 days). Despite the similarity in the departure dates to the wintering area between years, the arrival dates to the breeding colony were substantially different among years (Table 1). The averaged arrival dates to their colony occurred ca. 10 days earlier in 2009 compared to 2008 (13^th^ of September ±8.6 days and 3^rd^ of September ±6.7 days, t-test: *t_21_* = 3.75, P<0.005).

The day-length recorded when the birds started their post-breeding migration was very consistent between birds (Fig 2a, 12.56±0.16 hours) and years (2008: 12.50±0.13 and 2009: 12.63±0.16, *t*_21_ =  −1.92, P>0.05). The daylight durations at the initiation of the pre-breeding migration were the same as for the initiation of their post-breeding migration (12.53±0.11 hours, *t*_43_ = 0.139, P = 0.88) with no differences among individuals (Paired *t*-test: *t_21_* = 1.0304, P = 0.3145, Table 1).

### Night-time activity, migration dates and moon cycle

Similarly to day-time, night-time activity exhibited clear cycles and spectral analysis revealed two peaks at 182 and 365 days (Fig. 3b). During the night, Barau's petrels were also more active during the breeding season (10.5±9.3% of time spent on water, corresponding to a mean of 1.1 h ±0.9 h) than during the non-breeding season (Fig. 2b, 54.4±27.4% of time spent on water: *ie.* 6.8 h ±2.6 h) (*t*_747_ =  −30.85, P<0.005). These seasonal variations were greater than for day-time (Fig. 2). Spectral analysis also revealed a peak at 29.2 days (Fig. 3b) showing the existence of a cycle of 29.2 days affecting the at-sea activity during night. In fact, night-time activity was correlated to moon phases (*Pearson's correlation, r* =  −0.31, df = 750, P<0.005). Birds spent significantly less time resting on water during full moon where they spent up to 80% (maximum activity: 11.9 h per night) of their time in flight (Fig. 2b). Overall, birds responded more clearly to the lunar cycle during the non-breeding season (*Pearson's correlation, r =  −0.65,* df = 328, P<0.005) compared to the breeding season (*Pearson's correlation, r =  −0.44,* df = 420, P<0.005).

Although we found no clear correlation between initiation dates of pre- and post-breeding migrations and moon phases, the mean arrivals dates to the colonies coincided with the full moon in both years of the study (full moon: 15^th^ of September in 2008 and 5^th^ of September in 2009, and return date: 13^th^ of September ±8.6 days in 2008 and 3^rd^ of September ±6.7 days in 2009).

## Discussion

### Day-length effects

For the first time, bio-loggers were used to record daily the photoperiod perceived by a free-living migrant bird to study the influence of annual changes in daylength on their behavior. The at-sea activity and migration of Barau's petrels were strongly correlated to photoperiod, suggesting a clear proximal control of these behaviors by the duration of daylight. Firstly, the annual activity of birds exhibited seasonal changes in activity according to their annual cycle with a lower proportion of time on water during the breeding compared to the non-breeding stage. The increase in ecological constraints from a growing chick across the breeding season, as regular returns to the colony and higher energetic demands, may explain the higher flight activity during this period. This has been described for other seabirds species [20], [36], but surprisingly, we did not observe a clear bi-modal change in activity between breeding and non breeding periods. Variations of day-time activity were sinusoidal and correlated to seasonal changes of daylength (Fig. 2a). We thus hypothesize that the daily photoperiod received by birds could directly affect their behavior. The mechanism by which photoperiodic species actually measure day-length (or night length) is widely accepted to be based on circadian clock(s) [40]. Daily and seasonal changes in melatonin secretion encode respectively the time-of-day and the time-of-year information for birds [41], [42]. The duration of night-time melatonin secretion (melatonin peak) reflects the night length and hence, the day-length [43]; and change in the amplitude of melatonin secretion may reflects the season [42]. In consequence, seasonal photoperiod pressure acts on behavior and can increase the duration of flight event in birds [44], or change the daily pattern of locomotor activity in migrant birds [45]. Photoperiod is also known to promote extra feeding, fattening and migratory restlessness at appropriate dates for the population concerned [46], resulting in rhythmic behavior, metabolism, and physiological events [40]. However, this melatonin function is not universal in birds [47], change in amplitude of melatonin secretion could also be reduced during migration season despite constant photoperiod, resulting from physiological changed[45]. Future experimental studies are needed to validate our suggestion.

Secondly, migration dates of Barau's petrels were consistent between years and seem to be linked to photoperiod. Birds engaged their post- and pre-breeding migration trips when the day-length reached around 12.50 hours *i.e.* about 10 days after austral autumn equinox and 25 days before austral spring equinox, respectively. The slight inter-individual variations could be related to their own locations where environmental conditions are different in time. Overall, the effect of day-length (interacting with the circannual rhythm of each bird) is known to initiate the preparatory processes that precede each event [44]. A recent study suggests that birds are sensitive to small changes in photoperiod and may use subtle photoperiodic cues to time physiological responses [16], [48]. Birds retain a memory of photoperiod and so could perceive change in photoperiod [41], [49]. Around equinoxes, the daily rate of change in day-length is maximal, we thus assume that this may explain why these periods have been selected as synchronous migration periods. It has been suggested recently that the proper expression of migratory restlessness is also influenced by the intensity of ambient light that varies among the seasons [50]. Both the intensity and the length of daylight may thus influence times of migration. Despite low seasonal variations of photoperiod in the tropics, we suggest that a tropical seabird can measure slight photoperiodic changes and use these cues to initiate specific behaviors and time their breeding biology.

### Moon phase effects

Similarly to photoperiod, the moon phase clearly influenced the behavior of Barau's petrels. Their night-time at-sea activity followed repeated cycles according to lunar cycles (∼29 days). Petrels were more active during nights with higher moonlight intensity. Other studies have suggested that seabirds fly more frequently on nights around the full moon than on nights around the new moon [15], [34], [51]. For example, seabirds are more often caught by longline fisheries during bright moonlight than on nights with no moonlight [52]. The influence of the moon on Barau's petrel's at-sea activity is reduced during the breeding compared to the inter-nesting period (Fig. 2a), particularly around the new moon where petrels retain a high level of activity. We suggest that the higher energetic demands of the birds during the breeding season, and their need to return to the colony as often as possible require seabirds to maintain a higher foraging effort whatever the environmental conditions.

In wildlife, nocturnal illumination is an important factor influencing the predator/prey interaction [53], especially because moonlight generally improves the foraging efficiency of predators [54]. A first hypothesis that could explain the changes in Barau's petrel behavior with moon phase is that they forage for prey more frequently on nights with higher levels of moonlight intensity. Indeed, light levels during full moon are similar to the light levels at dawn and dusk [54], and birds could take advantage of this natural light source to increase their foraging success. A second non-exclusive hypothesis is that moonlight may change the abundance of prey available for seabirds and consequently their foraging activity. In marine environment, many endogenous cycles including movement, feeding, and reproduction in marine fishes and invertebrates, are cued to lunar phase [55]–[57]. Moonlight is known, for example, to affect the process of vertical migration of nekton [58], [59] that could increase the number of prey in the sub-surface layer where seabirds forage. Barau's petrels could therefore opportunistically increase their at-sea activity to take advantage of this higher prey abundance. A third hypothesis is that the moon light level could influence directly seabird behavior. Interestingly, as well as day-time activity being correlated to photoperiod, the Barau's petrels night-time activity followed the intensification of moonlight, reaching a maximum during full moon events. Accordingly, a recent study showed that light level at full moon modifies the melatonin secretion, and consequently potentially the behavior of seabirds [60].

Despite the year-to-year consistency in Barau's petrel initiation dates of pre- and post-breeding migrations and the clear influence of day-length to time these events, our results also suggest that moon phase may affect the arrival date at the colony after the pre-breeding migration. Such influence of the moon phase as been reported already for the sooty terns (*Onychoprion fuscatus*) breeding at Ascension island [11]. Authors have shown, with a dataset of 18 years, that the breeding cycle of this species corresponds to a period of ten lunar months [11]. Furthermore, they also demonstrated that each first arrival at the colony consistently happened during a full moon period. Interestingly, these field observations made 60 years ago and our results obtained with sophisticated electronic devices show very similar patterns suggesting that moon phase may act as synchronizer in many colonial seabirds.

Indeed, first arrival at the colony is crucial in the mating system of colonial animals like seabirds. Males and females are faithful and meet at the burrow to copulate [61]. Both sexes must therefore be as much as possible synchronized to maximize the mating opportunities and consequently, to optimize their fitness. It is therefore advantageous for seabirds to respond, not only to day-length but also to additional secondary factors such as moon cycle to time their life-history stages. In birds, secondary environmental factors are actually known to fine-tune the timing of various events to prevailing conditions [40], [62].

It would be very interesting to explore this idea that moon phase do synchronize seabirds breeding behavior to a wider range of seabird species under various environmental conditions.

To conclude, our study revealed intriguing and interesting results suggesting that both moon-phases and photoperiod affect the migration dates and the at-sea activity of Barau's petrels. These celestial cues may act on their biological life traits, triggering specific behaviors in space and time, and could be use as an annual calendar that best suit their needs. However, our results are only correlatives and do not allow to make direct causal inferences, and future experimental researches need to analyze underlying mechanisms. Finally, this novel use of data loggers provides new opportunities to study bird behavior under natural conditions. Currently, many species are tracked around the world and cross taxa studies could be very useful to investigate the influence of environmental cues on some fascinating behavioral decisions undertaken by free-living organisms.
